# Molecular docking and molecular dynamic simulation studies to identify potential terpenes against Internalin A protein of *Listeria monocytogenes*

**DOI:** 10.3389/fbinf.2024.1463750

**Published:** 2024-09-06

**Authors:** Deepasree K, Subhashree Venugopal

**Affiliations:** Department of Integrative Biology, School of Bio Sciences and Technology, Vellore Institute of Technology, Vellore, Tamil Nadu, India

**Keywords:** Internalin A, Lipinski’s rule, listeriosis, secondary metabolites, terpenes

## Abstract

**Introduction:**

Ever since the outbreak of listeriosis and other related illnesses caused by the dreadful pathogen *Listeria monocytogenes*, the lives of immunocompromised individuals have been at risk.

**Objectives and Methods:**

The main goal of this study is to comprehend the potential of terpenes, a major class of secondary metabolites in inhibiting one of the disease-causing protein Internalin A (InlA) of the pathogen via *in silico* approaches.

**Results:**

The best binding affinity value of −9.5 kcal/mol was observed for Bipinnatin and Epispongiadiol according to the molecular docking studies. The compounds were further subjected to ADMET and biological activity estimation which confirmed their good pharmacokinetic properties and antibacterial activity.

**Discussion:**

Molecular dynamic simulation for a timescale of 100 ns finally revealed Epispongiadiol to be a promising drug-like compound that could possibly pave the way to the treatment of this disease.

## 1 Introduction

Plants have been an ideal source of medicine since prehistoric times due to their beneficial effects on human health with minimum or no side effects. For this reason, medicinal plants and other plant-derived products are used extensively for treating various infectious diseases and ailments by the pharmaceutical industries. Medicinal plants are considered a ‘storehouse’ of an array of biologically active compounds with varied therapeutic applications (Pagare et al., 2015; Crozier et al., 2006; Tiwari and Rana, 2015; Yang et al., 2018). These plants possess anticancer, antibacterial, antiviral, analgesic and anti-inflammatory properties among their many other medicinal uses. More than 80% of the world’s developing population relies on traditional medical practices involving the usage of plants for their primary wellness as reported by the World Health Organization (WHO) (Bennett and Wallsgrove, 1994; Teoh and Teoh, 2016). The advancements in modern research has facilitated a significant surge in the study of plant metabolites throughout the last century. Secondary metabolites (SMs) are vital compounds that are typically produced by plants and have a distinct carbon skeleton framework. These metabolites are the end products of primary metabolites generated from the biosynthetic modifications such as methylation, glycosylation and hydroxylation. Although not directly involved in respiratory and photosynthetic metabolism, these metabolites assist plants in withstanding specific environmental conditions. In fact, the SMs are essential in defending the plant against potential threats like animals and various microbes. Moreover, certain plants utilize SMs to attract seed dispensers and pollinators, as well as signals to mediate the symbiotic relationship between plants and microorganisms (Aye et al., 2019; Twaij and Hasan, 2022). The side chains and structural composition certainly make SMs more complex. SMs can be categorized based on factors including their solubility in different solvents, chemical structure, biosynthetic pathways and composition. Terpenes (like sterols, carotenoids, plant volatiles, cardiac glycosides), phenolics (like coumarins, flavonoids, phenolic acids, lignin, tannins, stilbenes, lignans), nitrogen containing (like non-protein amino acids, alkaloids, cyanogenic glucosides) and sulfur containing compounds (like thionins, glutathione, phytoalexins, defensins) are some of the main groups belonging to SMs (Roba, 2020; Theis and Lerdau, 2003; Tetali, 2019).

Among the different phytochemicals, terpenes are the largest class of natural products with over 30,000 members that have been utilized for a wide range of applications such as flavoring, perfume, medicine, cosmetics, biofuels, food and beverages. They are the most diverse family that range in both structure and size. These volatile, unsaturated five-carbon cyclic molecules are composed of isoprene units, and the type of terpene that is formed varies depending on the number of isoprene units present (Costa et al., 2012). It is the hemiterpenes (1 isoprene unit) - dimethylallyl pyrophosphate (DMAPP) and isopentenyl pyrophosphate (IPP) that give rise to the many subclasses of terpenes. Terpene subclasses include mono- (2 isoprene units), sesqui- (3 isoprene units), Di- (4 isoprene units), sester- (5 isoprene units), tri- (6 isoprene units) and carotenoids (8 isoprene units). Numerous studies have elucidated the ability of terpenes to improve skin penetration, prevent inflammatory disorders, shield living organisms from biotic and abiotic stresses and also their antimicrobial property to combat infectious diseases (Croteau et al., 2000; Boncan et al., 2020; Cox-Georgian et al., 2019). The possible antibacterial potential of terpenes will be further explored in this work.

The food and economic sectors are being severely disrupted by the outbreak of foodborne illnesses and the subsequent death rates. Thus, public health initiatives have primarily focused on widely recognized foodborne diseases and pathogens in the food chain as a response to this growing concern (Altekruse et al., 1997; Motarjemi and Kiiferstein, 1997; Newell et al., 2010). Multiple factors contribute to the rise in foodborne diseases which include increase in immunocompromised individuals, lack of proper microbiological safety regulations before global food trading, transportation conditions that allow the pathogens to survive on the food and get to the consumers in a viable state, consumption of raw vegetables, meat and unpasteurized dairy products as well as temperature related changes favorable to the pathogen growth and production (Tauxe, 1997; Thakur et al., 2018). Listeriosis caused by *Listeria monocytogenes* (*L. monocytogenes*) is a systemic disease that arises from consuming contaminated food, notably ready-to-eat food and can be fatal to those with weak immune systems. Pregnant women are more vulnerable to this disease than the elderly people or infants, as several reports have stated 16%–17% of *Listeria monocytogenes* infections in this population (Matereke and Okoh, 2020; Ireton et al., 2021; Lecuit et al., 1997). Since this Gram-positive, psychrotolerant pathogen can pass through the blood brain and placental barriers, it often causes spontaneous miscarriages and meningoencephalitis in pregnant women. The pathogen’s tolerance to environmental stresses and sublethal doses of antibiotics or other antimicrobial drugs are significant factors to its antimicrobial resistance. Despite the advancements in the medical field to fight *L. monocytogenes*, the bacteria still pose a hazard to food safety as proved by the listeriosis outbreak that occurred in South Africa in 2017–2018. Of all the foodborne diseases, listeriosis has the highest death rates often ranging between 20% and 30% and sometimes even exceeds 50% (Bonazzi et al., 2009a; b; Lebrun et al., 1996). This pathogen expresses a wide range of virulence factors in order to proliferate and survive in the gastrointestinal system as well as to pass through the various host biological barriers. Internalins are one of the major virulence factors of *L. monocytogenes* that aids the pathogen to adhere and invade the host cell membrane. Among the internalins, the most researched ones are internalin A (InlA) and internalin B that play a vital role in its pathogenesis. InlA is an 88 kDa protein that comprises of a signal peptide at the N-terminus followed by 15 leucine rich repeats (LRR). An important structural component of InlA is an inter-repeat (IR) domain located downstream of the LRR domain that is essential for the binding of LRR region to its host cell receptor E-cadherin (Martin, 2003). This widely expressed and evenly distributed surface protein is covalently attached to the cell wall and upon binding to the E-cadherin receptor facilitates the pathogen’s internalization into the enterocytes and entry to the primary site of the host cell, i.e., the intestinal epithelial barrier. An LPTXG motif that anchors InlA to the bacterial cell wall is located at the C-terminal end of the protein followed by a sorting peptide (Lopes-Luz et al., 2021; Kathariou, 2002).

To date, no *in silico* investigations have been performed to predict the potential of terpenes in suppressing the activity of InlA protein. Thus, this study was intended to investigate the binding interactions of InlA with 80 terpenes through molecular docking and dynamic simulation approaches with an ultimate goal of identifying potential inhibitors of the virulence protein.

## 2 Materials and methods

### 2.1 Preparation of protein and ligands

**FIGURE 1:**
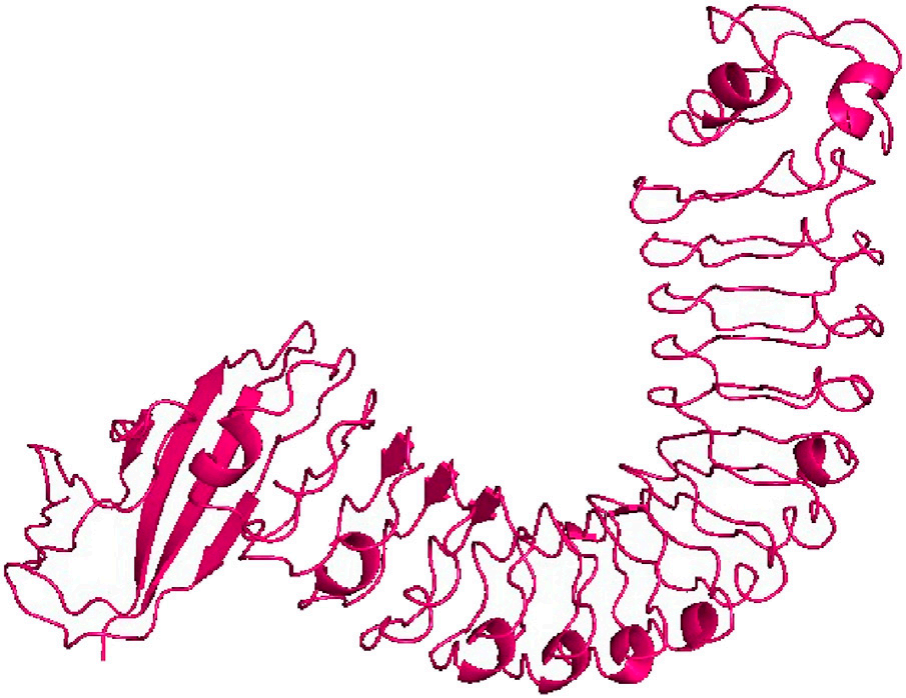
Crystal structure of InlA protein (PDB ID: 1O6T).

The 3D structure of InlA protein from *L. monocytogenes* with resolution of 1.60 Å (PDB ID: 1O6T) (Figure 1) was obtained in PDB format from the Research Collaboratory for Structural Bioinformatics (RCSB) Protein Data Bank (https://www.rcsb.org/, accessed 12 February 2024) (Berman et al., 2000). This protein was then subjected to preparation prior to docking using the AutoDock Tools 1.5.6 by eliminating water molecules, heteroatoms and adding polar hydrogens and Kollman charges. Finally, the prepared target was saved in pdbqt format (Morris et al., 2009).

In the case of ligands, 80 terpenes from different plant species with a range of medicinal properties were selected based on ‘Lipinski’s rule of five’ (Table 1) and the 3D structures of these compounds were obtained in SDF format from the PubChem database (https://pubchem.ncbi.nlm.nih.gov/, accessed12 February 2024) (Benet et al., 2016; Kim, 2021). The compounds were energy minimized using the steepest descent algorithm of Avogadro software and Open Babel program was further utilized to generate their pdbqt files (Hanwell et al., 2012; O'Boyle et al., 2011).

**TABLE 1:** Selected terpenes that are Lipinski compliant. The abbreviations HBD, HBA, Log P, RB and TPSA stands for Hydrogen bond donor, hydrogen bond acceptor, octanol-water partition coefficient, rotatable bonds and topological polar surface area.

Ligand no.	Compound name	Molecular weight (g/mol)	HBD	HBA	Log P	RB	TPSA (Å^2^)
1	Carvone	150.22	0	1	2.4	1	17.1
2	Aguerin B	330.4	1	5	1.9	3	72.8
3	Chlorojanerin	398.8	3	7	0.4	5	113
4	Janerin	362.4	2	7	0.1	4	106
5	Cynaropicrin	346.4	2	6	0.6	4	93.1
6	Britannin	366.4	1	7	1.9	4	99.1
7	Pulchellin	266.33	2	4	1.7	0	66.8
8	Zaluzanin C	246.30	1	3	1.3	0	46.5
9	Inuchinenolide C	366.4	1	7	1.9	4	99.1
10	Neopulchellin	266.33	2	4	1.7	0	66.8
11	Hemistepsin	346.4	2	6	0.6	4	93.1
12	Tetraneurin E	324.4	2	6	1.5	3	93.1
13	Bipinnatin	264.32	1	4	1.6	0	63.6
14	Athrolide B	394.5	1	7	2.9	5	99.1
15	Florilenalin	264.32	2	4	0.9	0	66.8
16	Annuolide C	246.30	1	3	1.5	0	46.5
17	Hysterin	308.4	1	5	2	3	72.8
18	Germacrene B	204.35	0	0	4.1	0	0
19	Alpha-Fenchene	136.23	0	0	3.1	0	0
20	Pinguisone	232.32	0	2	2.9	0	30.2
21	Furodysinin	216.32	0	1	3.9	0	13.1
22	Bisabolol	222.37	1	1	3.8	4	20.2
23	Hernandulcin	236.35	1	2	3.3	4	37.3
24	Lippidulcine A	252.35	2	3	1.6	4	57.5
25	Delobanone	236.35	1	2	3.1	4	37.3
26	Isoterpinolene	136.23	0	0	3.5	0	0
27	Artemisinin	282.33	0	5	2.8	0	54
28	Taurin	248.32	0	3	1.4	0	43.4
29	Santonin	246.30	0	3	2.3	0	43.4
30	Dehydrofarinosin	244.28	0	3	2.5	0	43.4
31	Yomogin	244.28	0	3	2.1	0	43.4
32	Encelin	244.28	0	3	2.5	0	43.4
33	Frullanolide	232.32	0	2	3.1	0	26.3
34	Tuberiferine	246.30	0	3	2.8	0	43.4
35	Traginone	178.27	0	1	2.4	3	17.1
36	Erivanin	266.33	2	4	1	0	66.8
37	Tanacetin	264.32	2	4	1	0	66.8
38	Rothin B	264.32	2	4	1.1	0	66.8
39	Arbusculin C	248.32	1	3	2.1	0	46.5
40	Telekin	248.32	1	3	2.1	0	46.5
41	Himachalene	204.35	0	0	3.9	0	0
42	Isolepidozene	204.35	0	0	4.1	0	0
43	Thujopsadiene	202.33	0	0	4.2	0	0
44	Axinysone C	250.33	1	3	2.2	1	46.5
45	Kanshone F	318.4	0	3	4	4	43.4
46	Ramarin B	234.33	1	2	2.4	1	37.3
47	Ramarin A	250.33	2	3	0.9	1	57.5
48	Lindestrene	214.30	0	1	3.7	0	13.1
49	Isosericenin	260.33	0	3	3.7	4	39.4
50	Isofischeric acid	246.30	1	3	3.3	3	50.4
51	Dihydroisochromolaenin	214.30	0	1	3.3	0	13.1
52	Euryopsonol	234.33	1	2	3.3	0	33.4
53	Petasalbin	234.33	1	2	3.7	0	33.4
54	Epispongiadiol	332.4	2	4	3.6	1	70.7
55	Cespitularin A	300.4	1	2	4.4	0	33.4
56	Tetradymol	234.33	1	2	3.4	0	33.4
57	Velatumin	280.32	3	5	0.4	1	90.9
58	Curcolonol	264.32	2	4	1.3	0	70.7
59	Furanofukinin	248.36	0	2	4.2	1	22.4
60	Zedoarofuran	264.32	2	4	1.3	0	70.7
61	Euryopsol	266.33	3	4	1.1	0	73.8
62	Citronellal	154.25	0	1	3	5	17.1
63	Cyperusol C	238.37	2	2	3.1	1	40.5
64	Rhombitriol	254.36	3	3	1.9	1	60.7
65	Oxyphyllol	238.37	2	2	3.1	1	40.5
66	Isodrimenediol	238.37	2	2	2.6	1	40.5
67	Junenol	222.37	1	1	4.2	1	20.2
68	Platambin	238.37	2	2	2.6	1	40.5
69	Arctiol	238.37	2	2	3.1	1	40.5
70	Pterocarpol	238.37	2	2	2.4	1	40.5
71	Rhombidiol	238.37	2	2	2.4	1	40.5
72	Sulphureuine D	254.36	3	3	2	2	60.7
73	Penicieudesmol A	238.37	2	2	3.6	1	40.5
74	Lairdinol A	238.37	2	2	3.1	1	40.5
75	Bonducellpin G	432.5	2	7	2.4	4	106
76	Lindenenone	228.29	0	2	2.7	0	30.2
77	Epicurzerenone	230.30	0	2	4	2	30.2
78	Scabequinone	260.28	0	4	3	1	56.5
79	Omphalone	188.18	0	3	1.1	1	47.3
80	Evodone	164.20	0	2	1.9	0	30.2

### 2.2 Molecular docking of InlA with the selected terpenes

AutoDock Vina program was used to dock all the 80 ligands against InlA protein inorder to gain an insight into the various interactions involved during the binding process (Trott and Olson, 2010). This program employs an efficient optimization technique that depends on a special scoring function (combination of empirical and knowledge-based approaches) and a gradient-based local search genetic algorithm to predict the binding modes thereby producing reliable information concerning the receptor and ligand interactions (Jaghoori et al., 2016; Sarkar et al., 2024). The binding affinities were estimated for each InlA-ligand complex. The protein was kept rigid and the ligands were flexible throughout the docking study. With the grid box centered at x = −15.289, y = −1.522 and z = 26.283 and dimensions set at points 126 × 82 × 126 separated by 1 Å, all the input files needed for docking were created in ADT 1.5.6. Based on the best docking scores, the top ten compounds from among the 80 terpenes were chosen for further interaction analysis. A compound with greater negative binding affinity value revealed its stronger binding to the target receptor (Rolta et al., 2022).

### 2.3 Visualization of binding interactions

Different interactions and the amino acid residues involved during the binding of InlA protein with the top 10 compounds were examined using the BIOVIA discovery studio (DS) visualizer and Ligplot+ software (Studio, 2008; Laskowski and Swindells, 2011). In particular, Ligplot+ was used to study the two-dimensional (2D) protein-ligand interactions involving hydrogen and hydrophobic interactions, and DS visualizer was used to investigate the different forms of hydrogen and hydrophobic bonding that were present in the complexes. Additionally, PyMOL software was also utilized for the three-dimensional (3D) visualization of receptor-ligand complexes (Yuan et al., 2017).

### 2.4 ADMET and biological activity prediction

The pharmacokinetic properties and the efficacy of the top 10 compounds obtained upon docking were computed by the admetSAR server (http://lmmd.ecust.edu.cn/admetsar2/, accessed 15 February 2024). The server predicts the carcinogenicity, blood-brain barrier penetration (BBB), Human Intestinal Absorption (HIA), subcellular localization, human oral bioavailability, toxicity and various other parameters of drugs or drug-like compounds that play a crucial role in the drug development process (Cheng et al., 2012). Additionally, the PASS (Prediction of Activity Spectra for Substances) online server (https://www.way2drug.com/passonline/, accessed 18 February 2024) was used to further explore the possible therapeutic activities of these compounds (Filimonov et al., 2014). This server estimates a compound’s expected activity spectrum as ‘probability of activity (Pa)’ and ‘probability of inactivity (Pi)’. Pa and Pi have values ranging from 0.000 to 1.000 and the compound activity is considered possible only if Pa > Pi (Chakraborty et al., 2016).

### 2.5 Molecular dynamic (MD) simulation and MM/GBSA binding free energy estimation

MD simulation of protein-ligand complexes with the best docking scores and the protein alone (InlA) was carried out using the GROMACS software (version 2023) for a 100 nanosecond (ns) timescale to gain an insight into their stability as well as the conformational behaviour upon ligand binding (Bauer et al., 2022). CHARMM27 forcefield of GROMACS and SwissParam server were used to generate the protein and ligand topologies (Bjelkmar et al., 2010; Bugnon et al., 2023). Both the protein and complex systems were further solvated in cubic box and neutralized by adding Na^+^/Cl^−^ ions. Post solvation and neutralization, using steepest descent algorithm the systems were energy minimized to eliminate the steric clashes and then equilibrated utilizing the NVT and NPT ensembles so as to maintain the temperature (300K) and pressure (1atm) of the systems (Berendsen et al., 1984; Andersen, 1980; Petersen, 1995). Ultimately, MD run was initiated for 100 ns and the root mean square deviation (RMSD), root mean square fluctuation (rmsf), radius of gyration (Rg), solvent accessible surface area (SASA) of the protein-ligand and protein systems were determined. Furthermore, the protein-ligand complexes were subjected to principal component analysis (PCA). Xmgrace software was used to obtain the 2D plots of all the aforementioned parameters (Turner, 2005).

Post MD simulation, MM/GBSA (Molecular Mechanics/Generalized Born Surface Area) binding free energy of the protein-ligand complexes with the best docking scores were estimated for the last 20 ns MD trajectory using the gmx_MMPBSA program (v1.6.0) (Valdés-Tresanco et al., 2021). In MM/GBSA method, the total binding free energy is determined by the following equation:

$$∆G_{\text{bind}}=G_{\text{complex}}\;-\;G_{\text{protein}}\;-\;G_{\text{ligand}}$$

$$∆G_{\text{bind}}=∆G_{\text{gas}}+∆G_{\text{sol}}\;-\;T∆S$$

where G_complex_ is the free energy of the protein-ligand complexes, while G_protein_ and G_ligand_ are the free energies of the protein and ligands. The conformational entropy upon ligand binding is represented by T∆S. ∆G_gas_ is the sum of internal energies (ΔE_int_ - bond, angle, dihedral), van der Waals energy (ΔE_vdW_) and electrostatic component of internal energy (ΔE_ele_). ∆G_sol_ is the combination of polar (∆E_GB_) and non-polar (∆E_SURF_) components of solvation energy.

## 3 Results

### 3.1 Lipinski rule evaluation of terpenes

All the selected compounds obeyed ‘Lipinski’s rule of 5’ without any violation suggesting their possibility of oral administration. According to this rule, a drug or a drug-like compound can be orally administered only if it satisfies the following criteria: molecular weight ≤500 g/mol, HBD ≤5, HBA ≤10 and LogP ≤ 5 (Walters, 2012; Saini et al., 2021). The molecular weight of all the terpenes varied from 136.23 to 398.8 g/mol. Alpha-Fenchene and Isoterpinolene had the lowest molecular weight of 136.23 g/mol while the highest value of 398.8 g/mol was shown by Chlorojanerin. Moreover, the HBDs and the HBAs of all the compounds ranged from 0 to 3 and 0 to 7, respectively. The lowest logP value of 0.1 was noted for Janerin whereas Cespitularin A had the maximum logP value of 4.4. These observations collectively demonstrate the potential of the chosen terpenes in oral delivery.

### 3.2 Molecular docking analysis of protein with the terpenes

**FIGURE 2:**
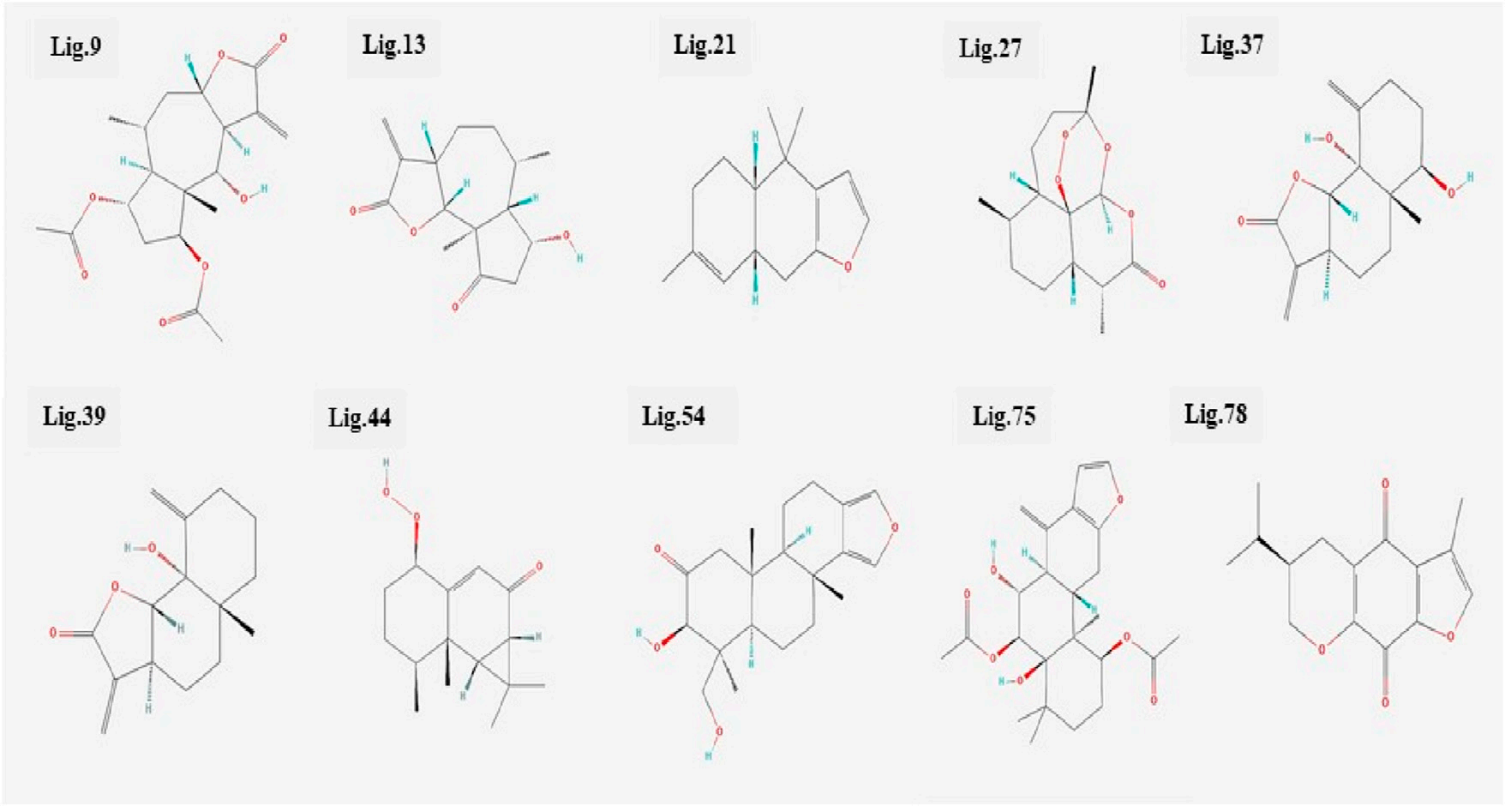
Structures of the top 10 terpenes.

All the 80 terpenes were blindly docked against the InlA protein with the grid box covering the entire protein surface. Additionally, a reference ligand Ampicillin, which is one of the most widely used antibiotic to treat illnesses caused by *L. monocytogenes,* was also docked against the virulence protein in order to determine whether the terpenes were more or less effective compared to the drug. We observed exceptional binding affinity values for each protein-ligand complexes and the top 10 compounds (Figure 2) with the highest scores were finalised for further analysis. The important amino acid residues and the different interactions involved in the binding of the best 10 ligands with the InlA protein from DS visualizer and Ligplot+ software are displayed in table 2. The binding affinity values obtained for all the 80 terpenes and Ampicillin are highlighted in table 3.

**TABLE 2:** Top 10 terpenes with the best docking scores and their interacting residues.

Ligand no.	Ligand name	Binding affinity (kcal/mol)	Amino acid residues involved (discovery studio visualizer)					Amino acid residues involved (Ligplot+)	
			H-bond	Pi-sigma	Pi-alkyl	Alkyl	Others	H-bond	Hydrophobic
9	Inuchinenolide C	−9.0	Ser429	—	—	Val422	Ser429 (Unfavourable acceptor-acceptor bond)	Ser429 (3)	Asp457, Val428, Asn427, Lys425
13	Bipinnatin	−9.5	Ser173,Asp213	Phe150	Phe150 (2)	—	—	Ser173, Ser172, Asp213	Asn129, Asn151, Phe150
21	Furodysinin	−8.9	Ser172 (pi-donor)	—	Phe150 (4)	—	—	—	Ser173, Asp213, Glu170, Phe150, Ser172
27	Artemisinin	−9.1	Ser172	Phe150	Phe150	—	—	—	Ser173, Ser172, Phe150, Glu170, Asp213
37	Tanacetin	−8.9	Ser172 (2) Ser192	—	Phe150	—	—	Ser172, Ser173	Asp213, Phe150, Glu170, Ser192
39	Arbusculin C	−9.0	Ser173, Ser172, Ser192	—	Phe150	—	—	Ser172, Ser173	Asp213, Ser192, Glu170, Phe150
44	Axinysone C	−8.8	Ser215	—	Phe150 (3)	—	—	—	Ser173, Ser215, Ser172, Asp213, Phe150
54	Epispongiadiol	−9.5	Ser172 (carbon h-bond), Asn129 ((2) 1 is pi- donor)	—	Phe150 (3)	—	—	Asn129, Glu170	Asn151, Phe150, Ser172
75	Bonducellpin G	−9.1	Asn151, Asn129, Ser173, Ser215, Asp213	—	Phe150	—	Phe150 (Pi-Pi stacked bond)	Ser173, Asn129	Ser215, Ser216, Ser172, Phe150, Glu170
78	Scabequinone	−9.0	Ser173, Ser215 (pi-donor)	—	Phe150	—	Asp213 (Pi-anion bond)	Ser173	Ser215, Thr237, Asp213, Ser172, Glu170, Phe150

**TABLE 3:** Docking scores of all the 80 terpenes and Ampicillin.

Sl.No.	Ligands	Binding affinity (kcal/mol)
1	Carvone	−6.9
2	Aguerin B	−8.6
3	Chlorojanerin	−8.7
4	Janerin	−8.7
5	Cynaropicrin	−8.5
6	Britannin	−8.3
7	Pulchellin	−8.2
8	Zaluzanin C	−7.7
9	Inuchinenolide C	−9.0
10	Neopulchellin	−8.0
11	Hemistepsin	−8.7
12	Tetraneurin E	−8.1
13	Bipinnatin	−9.5
14	Athrolide B	−8.6
15	Florilenalin	−7.9
16	Annuolide C	−8.4
17	Hysterin	−8.0
18	Germacrene B	−7.9
19	Alpha-Fenchene	−6.1
20	Pinguisone	−8.0
21	Furodysinin	−8.9
22	Bisabolol	−8.1
23	Hernandulcin	−7.8
24	Lippidulcine A	−8.3
25	Delobanone	−8.7
26	Isoterpinolene	−7.3
27	Artemisinin	−9.1
28	Taurin	−8.3
29	Santonin	−8.4
30	Dehydrofarinosin	−8.5
31	Yomogin	−8.2
32	Encelin	−8.6
33	Frullanolide	−8.0
34	Tuberiferine	−8.2
35	Traginone	−7.6
36	Erivanin	−8.3
37	Tanacetin	−8.9
38	Rothin B	−8.6
39	Arbusculin C	−9.0
40	Telekin	−8.7
41	Himachalene	−7.6
42	Isolepidozene	−7.8
43	Thujopsadiene	−7.6
44	Axinysone C	−8.8
45	Kanshone F	−7.9
46	Ramarin B	−7.5
47	Ramarin A	−8.4
48	Lindestrene	−8.3
49	Isosericenin	−6.5
50	Isofischeric acid	−7.7
51	Dihydroisochromolaenin	−7.6
52	Euryopsonol	−8.1
53	Petasalbin	−8.4
54	Epispongiadiol	−9.5
55	Cespitularin A	−7.9
56	Tetradymol	−7.9
57	Velatumin	−7.8
58	Curcolonol	−7.9
59	Furanofukinin	−7.4
60	Zedoarofuran	−8.7
61	Euryopsol	−8.7
62	Citronellal	−5.4
63	Cyperusol C	−8.0
64	Rhombitriol	−7.8
65	Oxyphyllol	−8.1
66	Isodrimenediol	−7.4
67	Junenol	−7.9
68	Platambin	−7.8
69	Arctiol	−7.2
70	Pterocarpol	−8.4
71	Rhombidiol	−8.2
72	Sulphureuine D	−7.6
73	Penicieudesmol A	−8.1
74	Lairdinol A	−7.7
75	Bonducellpin G	−9.1
76	Lindenenone	−7.8
77	Epicurzerenone	−6.5
78	Scabequinone	−9.0
79	Omphalone	−7.9
80	Evodone	−6.7
Reference Ligand	Ampicillin	−8.7

**FIGURE 3:**
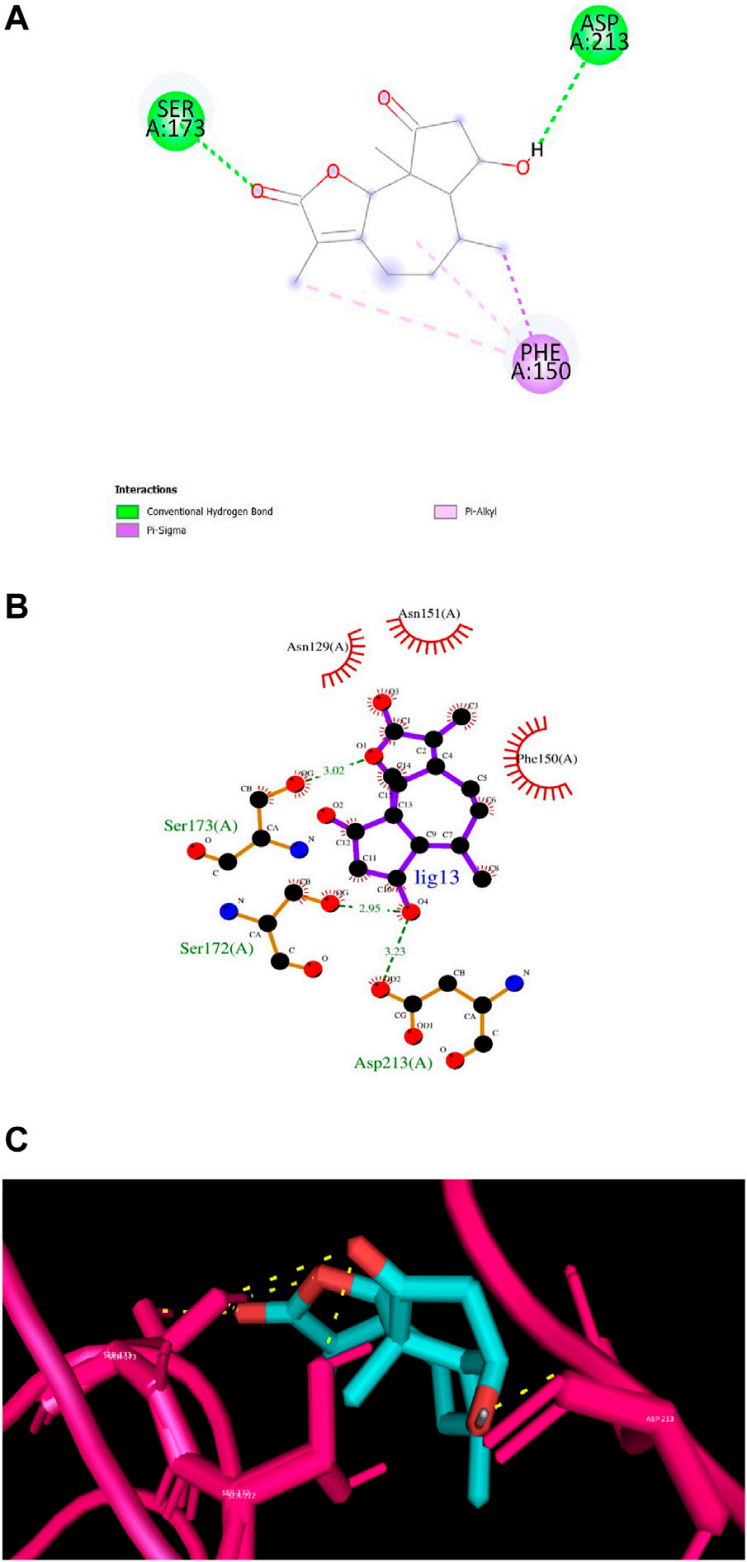
**(A)** and **(B)** 2D images of Bipinnatin interacting with InlA protein observed in discovery studio and Ligplot+. Green dotted lines indicate the hydrogen bonds involved while the red pockets containing amino acid residues reveal the residues involved in hydrophobic interactions **(C)** 3D view of Bipinnatin-InlA complex. Yellow dotted lines represent the hydrogen bonds formed during the binding process.

**FIGURE 4:**
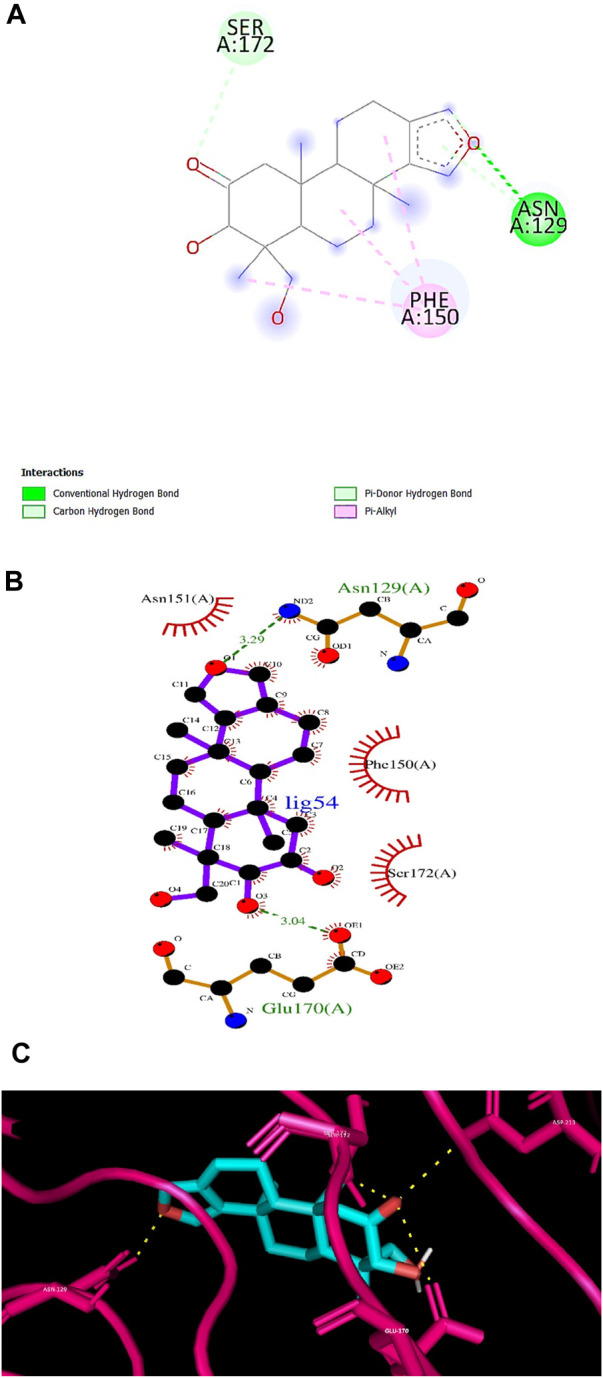
**(A)** and **(B)** 2D images of Epispongiadiol interacting with InlA protein observed in discovery studio and Ligplot+. **(C)** 3D view of Epispongiadiol-InlA complex.

According to numerous studies, higher negative binding affinity values often imply a ligand’s increased capacity to inhibit the disease-causing target protein (Deepasree and Subhashree, 2023; Sahu and Pattanayak, 2019; Islam et al., 2021; Correa-Basurto et al., 2015). The top 10 terpenes had varied binding affinities ranging from −8.8 to −9.5 kcal/mol. Among all the selected terpenes, Bipinnatin (lig.13) and Epispongiadiol (lig.54) were found to have displayed the best interaction with InlA protein with a binding affinity value of −9.5 kcal/mol followed by Artemisinin and Bonducellpin G showing the next highest docking score of −9.1 kcal/mol. In DS visualizer, Bipinnatin displayed 2 hydrogen bonds with Ser173 and Asp213 amino acid residues of InlA along with 3 hydrophobic interactions (2 pi-alkyl and 1 pi-sigma bonds) with Phe150 (Figure 3A). Similarly, the ligand13-InlA interaction analysis with Ligplot+ revealed a maximum of 3 hydrogen bonds to Ser172, Ser173 and Asp213 residues formed at a distance of 2.95 Å, 3.02 Å and 3.23 Å. Here, Asn129, Phe150 and Asn151 had hydrophobic interactions with Bipinnatin (Figure 3B). The 3D representation of ligand13-InlA complex is shown in Figure 3C. In the case of Epispongiadiol, the ligand showed 1 conventional and 1 pi-donor hydrogen bond with Asn129, 1 carbon hydrogen bond with Ser172 and 3 pi-alkyl bonds with Phe150 in DS visualizer (Figure 4A) while 2 hydrogen bonds to Asn129 and Glu 170 (formed at a distance of 3.29 Å and 3.04 Å) were noted in Ligplot+ (Figure 4B). Moreover, the compound had hydrophobic interactions with Asn151, Phe150 and Ser172 residues of the virulence protein. Figure 4C depicts the 3D view of ligand54-InlA complex.

**FIGURE 5:**
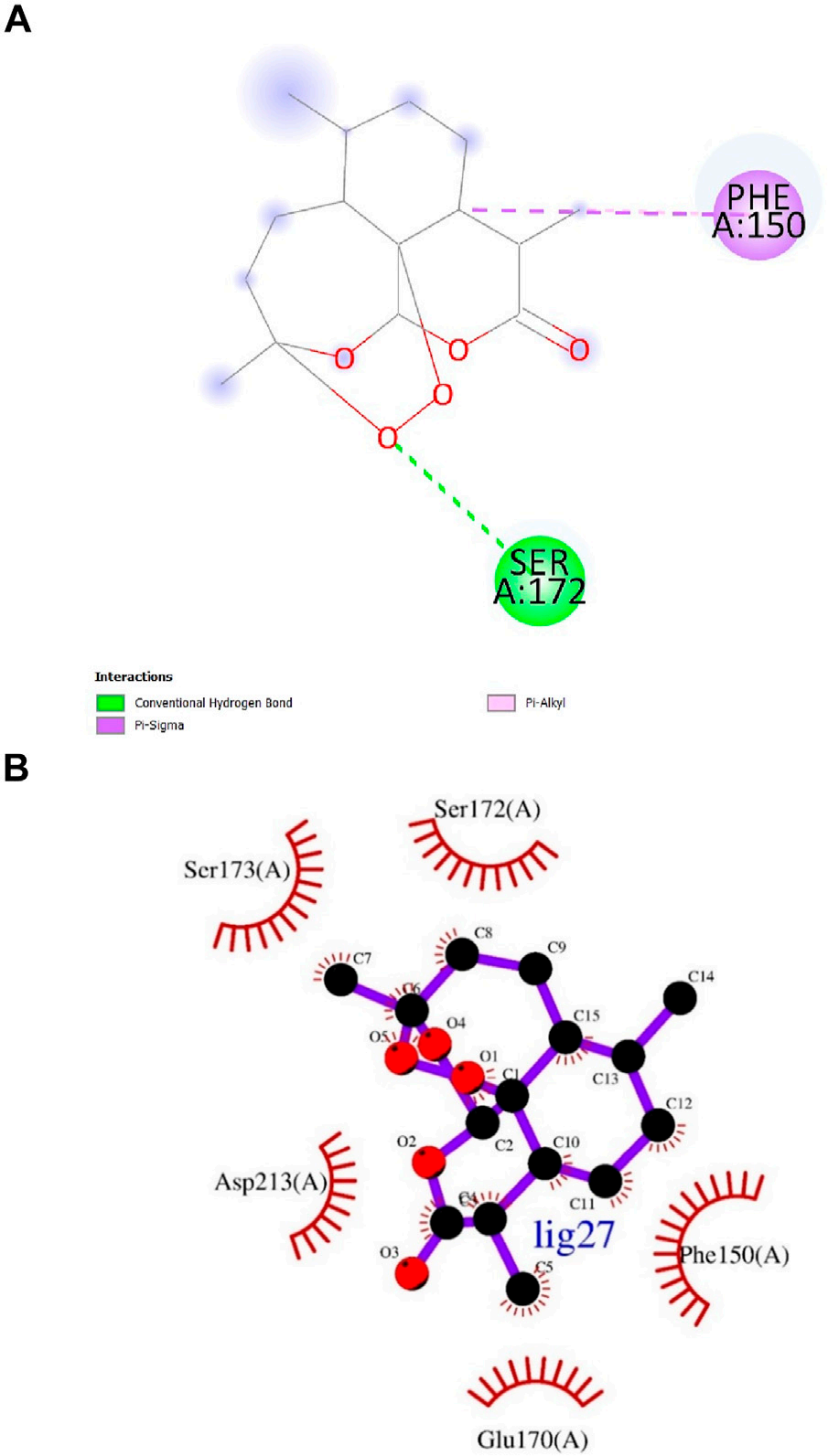
**(A)** and **(B)** 2D images of Artemisinin interacting with InlA protein observed in discovery studio and Ligplot+.

**FIGURE 6:**
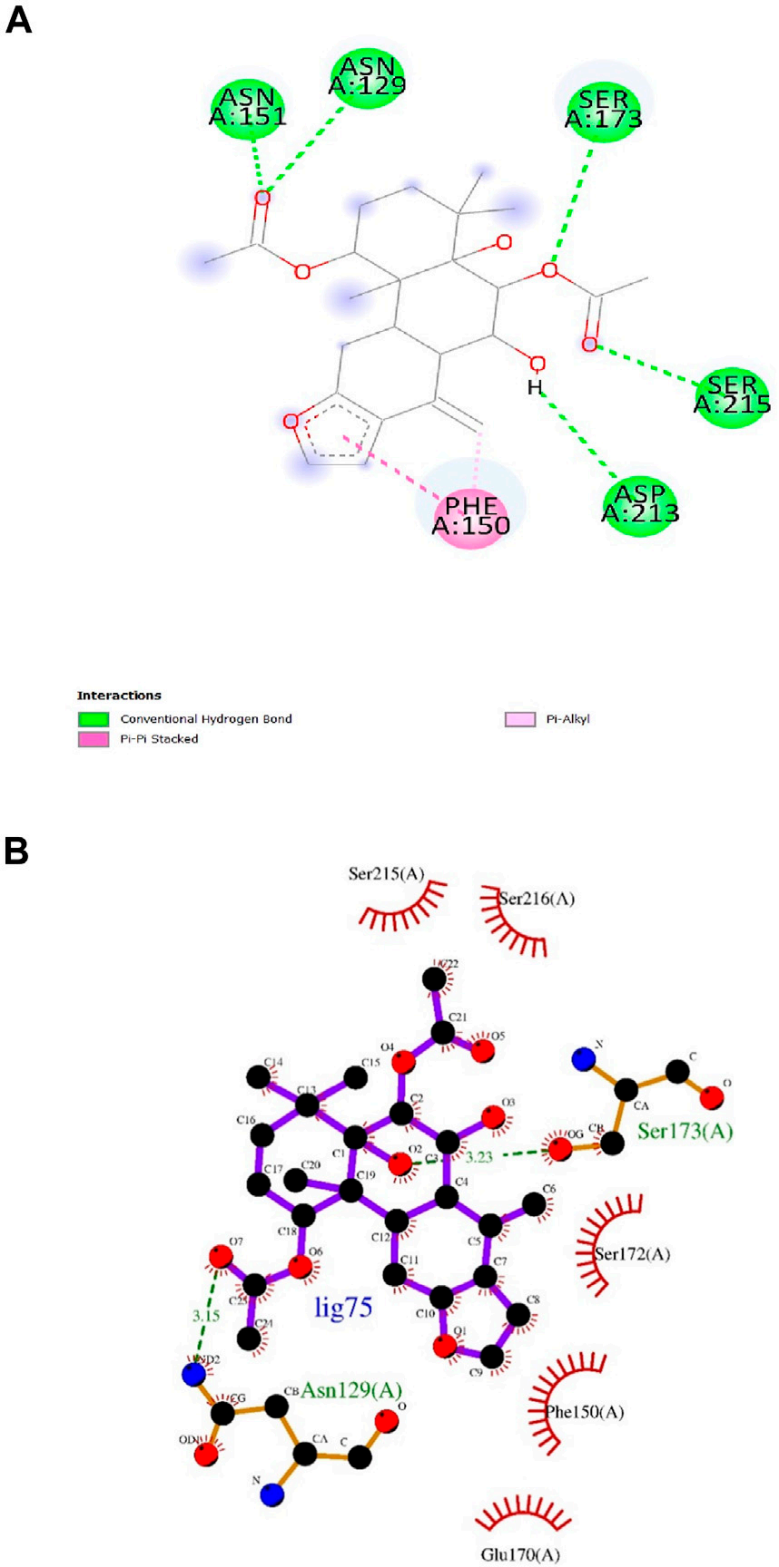
**(A)** and **(B)** 2D images of Bonducellpin G interacting with InlA protein observed in discovery studio and Ligplot+.

Artemisinin (ligand 27) that exhibited the next best affinity value (−9.1 kcal/mol) had 1 hydrogen bond with Ser172, 1 pi-alkyl and 1 pi-sigma bond with Phe150 residue of the protein (DS visualizer- Figure 5A). No hydrogen bonds were revealed upon the visualization of ligand 27-InlA complex in Ligplot+ and only hydrophobic interactions to Ser173, Ser172, Phe150, Glu170 and Asp213 were identified (Figure 5B). Also, Bonducellpin G (ligand 75) sharing the same value as that of ligand 27 possessed 5 hydrogen bonds with residues including Asn151, Asn129, Ser173, Ser215 and Asp213 as well as 1 pi-alkyl bonding with Phe150. The ligand also had an additional pi-pi stacked bonding with Phe150 residue (DS visualizer- Figure 6A). Figure 6B (Ligplot+) shows that ligand 75 formed hydrogen bonds with Asn129 and Ser173 each at a distance of 3.15 Å and 3.23 Å. Moreover, this compound had hydrophobic interactions with residues, namely, Ser215, Ser216, Ser172, Phe150 and Glu170.

**FIGURE 7:**
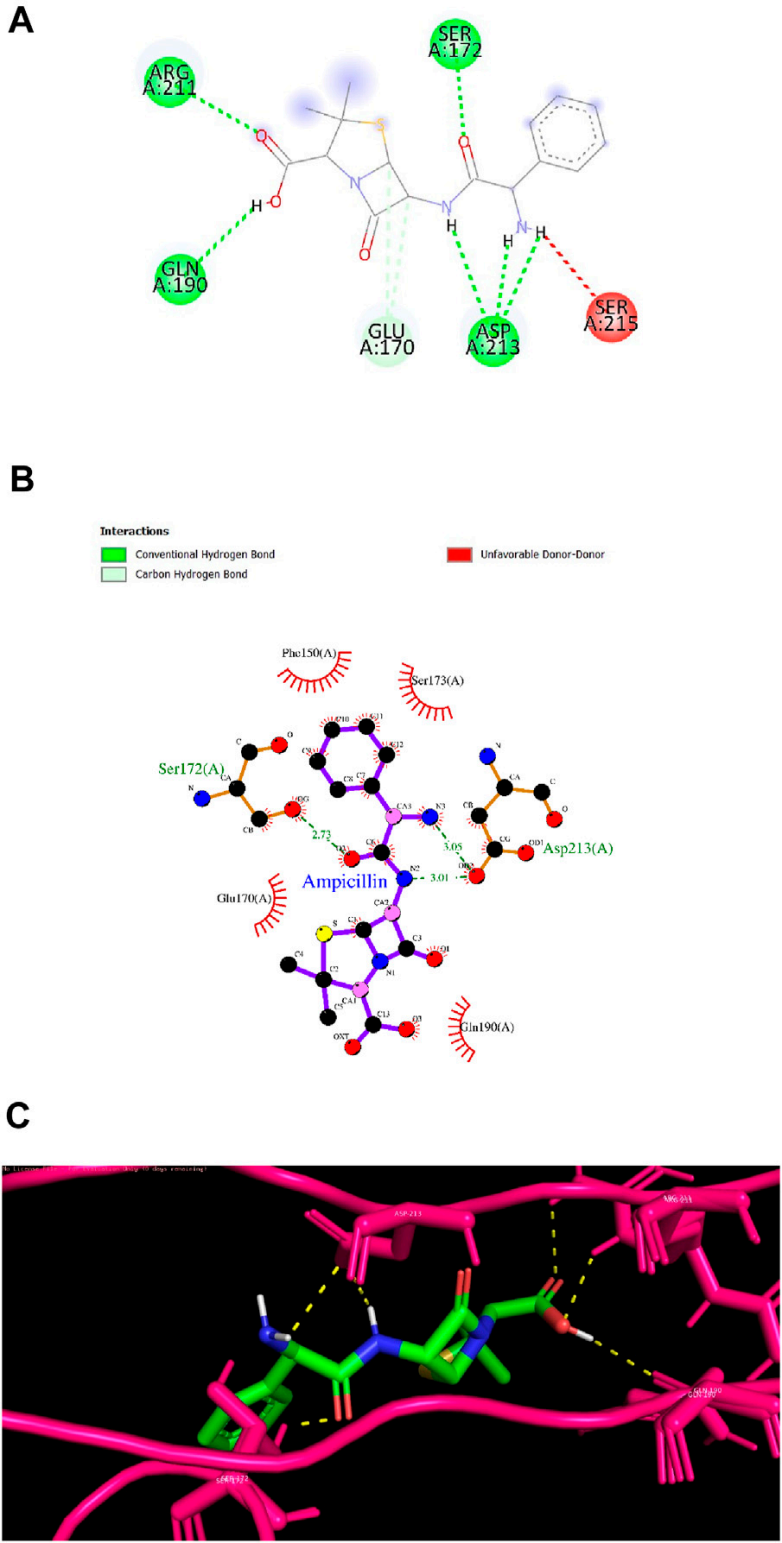
**(A)** and **(B)** 2D images of Ampicillin interacting with InlA protein observed in discovery studio and Ligplot+. **(C)** 3D view of Ampicillin-InlA complex.

Ligand 9 (Inuchinenolide C), ligand 39 (Arbusculin C), and ligand 78 (Scabequinone) had a docking score of −9.0 kcal/mol respectively. Out of the best 10 compounds, Axinysone C (ligand 44) showed the lowest binding affinity value of −8.8 kcal/mol with InlA protein indicating its weak interaction with the target protein when compared to the other compounds in the top 10 list. In contrast to the top 10 terpenes with the best docking scores, Ampicillin displayed a remarkably low binding affinity value of −8.7 kcal/mol. This result reveals that the top 10 terpenes exhibited stronger interaction with InlA in comparison to Ampicillin, pointing to the terpenes’ potential to be more bactericidal than the drug. Figures 7A, B (2D images) and Figure 7C (3D images) depict the various interactions involved between Ampicillin and InlA. Therefore, based on the docking results, Bipinnatin and Epispongiadiol with the highest docking scores could possibly act as potential antibacterial agents that can suppress the virulence protein InlA of *L. monocytogenes*. Furthermore, the key residues that were observed predominantly in the docked complexes were Ser172, Ser173, Phe150 and Asp213. This may also highlight the vital role played by these residues in inhibiting the protein upon binding with the antibacterial drugs thereby turning the virulence protein inactive.

### 3.3 ADMET and biological activity analysis

Post docking, the 10 terpenes were subjected to ADMET and biological activity analysis for a deeper understanding of their pharmacokinetic profiles and wide spectrum of therapeutic functions particularly the antibacterial activity. Some of the significant pharmacokinetic characteristics and therapeutic activities observed for the top 10 terpenes are shown in tables 4, 5.

**TABLE 4:** Pharmacokinetic properties of the top 10 terpenes.

Ligand no.	Compound	Human intestinal absorption (HIA)	P-glycoprotein (P-gp) inhibitor	Caco-2 permeability	Blood brain barrier penetration (BBB)	Carcinogenicity	Acute oral toxicity	Subcellular localization
9	Inuchinenolide C	Yes (+)	No (−)	No (−)	Yes (+)	No (−)	II	Mitochondria
13	Bipinnatin	Yes (+)	No (−)	Yes (+)	Yes (+)	No (−)	II	Mitochondria
21	Furodysinin	Yes (+)	No (−)	Yes (+)	Yes (+)	No (−)	III	Plasma Membrane
27	Artemisinin	Yes (+)	No (−)	Yes (+)	Yes (+)	No (−)	IV	Mitochondria
37	Tanacetin	No (−)	No (−)	Yes (+)	No (−)	No (−)	IV	Mitochondria
39	Arbusculin C	No (−)	No (−)	Yes (+)	No (−)	No (−)	III	Mitochondria
44	Axinysone C	Yes (+)	No (−)	Yes (+)	Yes (+)	No (−)	III	Mitochondria
54	Epispongiadiol	Yes (+)	No (−)	Yes (+)	Yes (+)	No (−)	III	Mitochondria
75	Bonducellpin G	Yes (+)	No (−)	No (−)	Yes (+)	No (−)	III	Mitochondria
78	Scabequinone	Yes (+)	No (−)	Yes (+)	Yes (+)	No (−)	III	Mitochondria

**TABLE 5:** Different biological activities exhibited by the top 10 terpenes.

Ligand no.	Compound	Activity	Pa	Pi
9	Inuchinenolide C	Antineoplastic Antiprotozoal Antileukemic Immunosuppressant Antifungal Antiinflammatory Antioxidant	0.951 0.928 0.704 0.720 0.628 0.553 0.254	0.004 0.003 0.005 0.014 0.016 0.042 0.035
13	Bipinnatin	Antineoplastic Antieczematic Antiprotozoal Antileukemic Immunosuppresant Antiinflammatory Antifungal Antibacterial Antiviral	0.936 0.896 0.787 0.761 0.763 0.700 0.567 0.434 0.386	0.004 0.005 0.005 0.005 0.009 0.016 0.022 0.024 0.108
21	Furodysinin	Antieczematic Dementia treatment Antifungal Antiinflammatory Antiviral Antiprotozoal Antineoplastic	0.708 0.490 0.403 0.415 0.339 0.309 0.372	0.043 0.017 0.049 0.088 0.178 0.094 0.115
27	Artemisinin	Antiprotozoal Antineoplastic Antiparasitic Antifungal Antileukemic Immunosuppressant Antiviral Antibacterial	0.992 0.853 0.857 0.793 0.806 0.825 0.639 0.214	0.001 0.007 0.002 0.005 0.004 0.003 0.001 0.005
37	Tanacetin	Antineoplastic Dementia treatment Antileukemic Antifungal Antibacterial Antiviral Antiprotozoal	0.860 0.529 0.506 0.457 0.332 0.375 0.245	0.006 0.010 0.014 0.038 0.049 0.122 0.069
39	Arbusculin C	Antineoplastic Antiprotozoal Antiinflammatory Antileukemic Antifungal Antibacterial Antiviral Dementia treatment Immunosuppressant	0.947 0.768 0.564 0.561 0.442 0.331 0.385 0.513 0.464	0.004 0.006 0.040 0.010 0.041 0.049 0.009 0.012 0.050
44	Axinysone C	Immunosuppressant Antineoplastic Antieczematic Antileukemic Dermatologic Antiseborrheic Dementia treatment	0.601 0.595 0.600 0.267 0.559 0.506 0.391	0.029 0.046 0.086 0.053 0.021 0.060 0.057
54	Epispongiadiol	Antineoplastic Antiinflammatory Antiviral Antifungal Antibacterial Antipruritic Dementia treatment Immunosuppressant	0.905 0.854 0.658 0.430 0.343 0.577 0.712 0.550	0.005 0.005 0.009 0.044 0.045 0.019 0.007 0.007
75	Bonducellpin G	Antineoplastic Hepatoprotectant Antipruritic Antiprotozoal Antifungal Immunosuppressant Antibacterial	0.732 0.689 0.560 0.488 0.455 0.416 0.246	0.021 0.008 0.022 0.026 0.038 0.062 0.085
78	Scabequinone	Vasoprotector Hepatoprotectant Antiinflammatory Antifungal Antibacterial Antileukemic Antioxidant Insulin promoter	0.691 0.577 0.574 0.459 0.379 0.378 0.365 0.399	0.011 0.014 0.037 0.038 0.035 0.029 0.015 0.064

One of the biggest challenges faced during oral drug development is assessing the drug’s capability to cross the intestinal epithelial barrier that estimates the rate and extent to which the drug is absorbed into the human body eventually impacting its bioavailability (Rudrapal et al., 2022; Aja et al., 2021; Qaddir et al., 2017). For this reason, both HIA and Caco-2 intestinal cell permeability of the compounds were estimated. Except for Tanacetin and Arbusculin C, the remaining terpenes exhibited positive response for HIA indicating that each of those terpenes can undergo intestinal absorption upon oral administration. Also, Inuchinenolide C and Bonducellpin G did not show caco-2 permeability while all other terpenes had positive response for the same. P-glycoprotein, an ABC transporter is a protein that actively engages in a number of biological processes including drug absorption, metabolism, distribution and excretion thereby detoxifying and protecting the body from harmful substances. It was discovered that none of the 10 compounds inhibited this protein. With the exception of Tanacetin and Arbusculin C, the remaining terpenes revealed BBB penetration. Moreover, all the terpenes were observed to be non-carcinogens. The subcellular localization of the terpenes were found to be in the mitochondria except for Furodysinin in plasma membrane. In the case of acute oral toxicity, the terpenes were categorized into four levels-mainly category I, II, III and IV based on their LD50 values. Category I consists of compounds with LD50 ≤ 50 mg/kg while category II has compounds with LD50 > 50 mg/kg but less than 500 mg/kg. Compounds with LD50 values higher than 500 mg/kg but less than 5,000 mg/kg are grouped into category III and those with LD50 value greater than 5,000 mg/kg comes under category IV. Generally, compounds that fall in category III and IV are often considered non-toxic while II is slightly toxic and category I compounds are highly toxic (Guan et al., 2019). In this study, none of the terpenes belonged to category I for acute oral toxicity. However, Inuchinenolide C and Bipinnatin displayed category II toxicity indicating their slightly toxic nature. Aside from Artemisinin and Tanacetin that belonged to category IV, all other terpenes were placed in category III. Thus, the top 10 compounds were predicted to have satisfactory ADMET findings for the major parameters.

The biological activities possessed by the 10 terpenes were further explored using the PASS web server. Antineoplastic, antiprotozoal, immunosuppressant, antiinflammatory, antifungal, antiviral and antileukemic activities were some of the predominantly expressed functions of the 10 terpene compounds obtained from the server and the Pa value was significantly higher than Pi in all the activity predictions. Furthermore, all the terpenes except for Inuchinenolide C, Furodysinin and Axinysone C exhibited antibacterial activity supporting the core aim of the study.

### 3.4 MD simulation analysis

Bipinnatin (ligand 13) and Epispongiadiol (ligand 54) showcased the highest binding affinities (−9.5 kcal/mol) based on the docking data obtained and hence MD simulation was performed for the complexes of these compounds with InlA protein as well as the apo form of InlA over a 100 ns timescale. Here, the RMSD, RMSF, Rg and SASA of the complexes and the apo protein were computed as a function of time inorder to monitor the variations in their stability, conformation and the amino acid residue interactions (Joshi et al., 2021; Surti et al., 2020; Nagamalla et al., 2022).

#### 3.4.1 RMSD analysis

**FIGURE 8:**
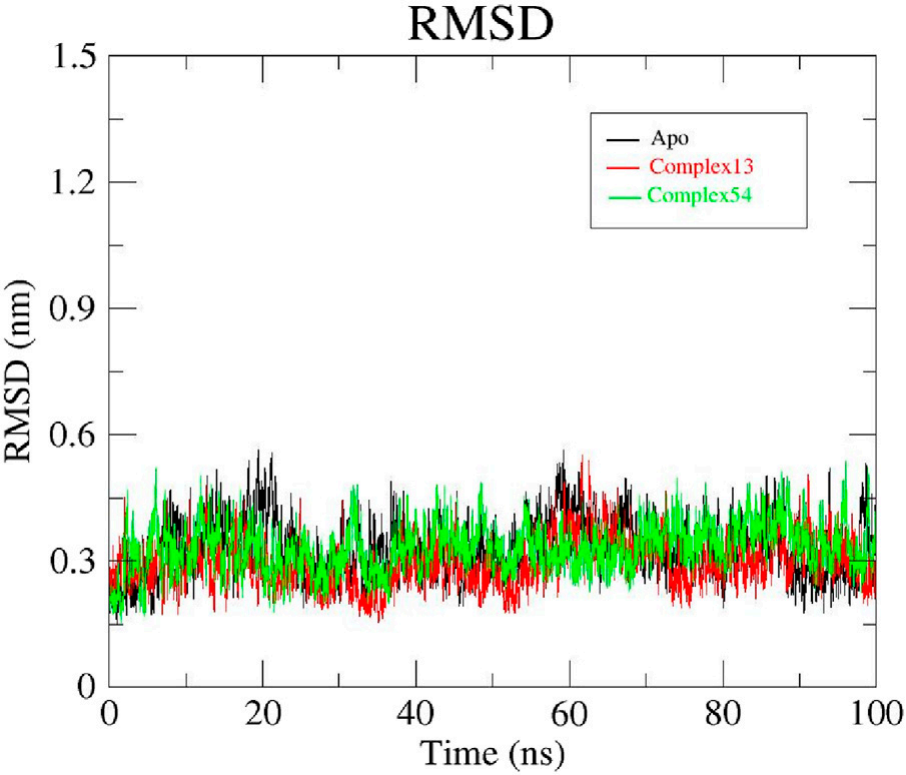
RMSD plots of Apo protein (black), Bipinnatin-InlA complex (red) and Epispongiadiol-InlA complex (green).

The RMSD was assessed to understand the stability of both the complex systems and apo protein that provides information on the degree of protein deviation from its native conformation upon ligand binding (Mani et al., 2023; Rampogu et al., 2022). The backbone RMSD plots of ligand13-InlA, ligand54-InlA and apo form are depicted in Figure 8. The apo protein started off with an RMSD value of 0.25 nm and then attained stability at 0.3 nm after a time period of 65 ns. This value was maintained till the end of 100 ns MD run. The Bipinnatin-InlA complex (complex13), displayed stability upto 20 ns at 0.3 nm RMSD but then significant deviations were seen until 70 ns at 0.45 nm. After 70 ns, the complex regained stability at the initial backbone RMSD value of 0.3 nm and remained stable with the same RMSD value until the 100 ns timescale. On the other hand, Epispongiadiol-InlA complex (complex54) was found to be stable after 50 ns maintaining a backbone RMSD value of 0.3 nm. A slight decrease in RMSD was noticed at 90 ns. Thus, ligand54- InlA complex was observed to be more stable than ligand13-InlA complex and apo protein according to the obtained RMSD results.

#### 3.4.2 RMSF analysis

**FIGURE 9:**
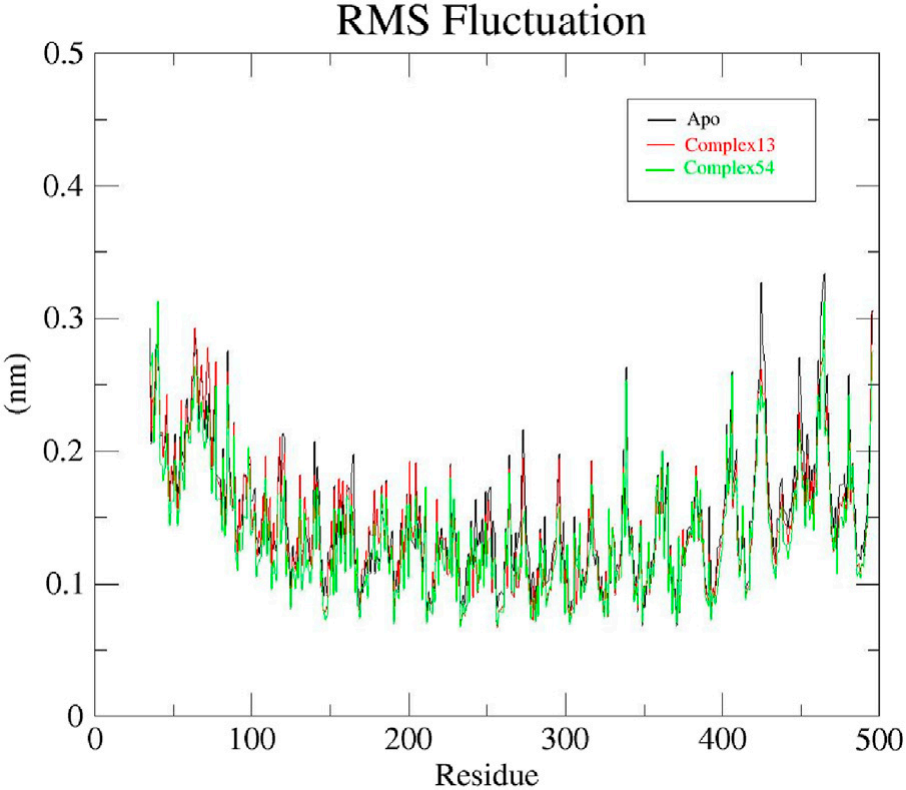
RMSF plots of Apo protein (black), Bipinnatin-InlA complex (red) and Epispongiadiol-InlA complex (green).

To gain a better understanding of the fluctuations in protein atoms or residues resulting from ligand interaction, RMSF analysis was carried out for all the 3 systems: apo protein, Bipinnatin-InlA and Epispongiadiol- InlA complexes. The presence of more flexible regions (turns, loops) are indicated by higher backbone RMSF values while lower value confirms the presence of secondary structures (helices or sheets) (Bhatt et al., 2021; Shahlaei et al., 2011; Krushna et al., 2017). The initial RMSF of apo protein was recorded to be 0.29 nm and as the 100 ns run proceeded, maximum fluctuations were observed at 0.33 nm between residues 420–440 and 450–480. Similarly, the Bipinnatin-InlA complex also began with an RMSF value of 0.29 nm and higher fluctuations were visible at 0.3 nm between residues 490 and 500. Minimum fluctuations with an average RMSF value of 0.2 nm were noted between the start and end of the 100 ns trajectory. The starting RMSF value of Epispongiadiol-InlA complex was noted to be 0.31 nm and higher variations were observed between residues 450–480 at 0.3 nm. Hence, from the above results it can be inferred that both ligand13-InlA and ligand54-InlA complexes did not exhibit much fluctuations from the apo protein (Figure 9) indicating the presence of more secondary structures than flexible regions in all the systems. This can further reveal that the protein has undergone less structural changes as a result of ligand binding.

#### 3.4.3 Rg analysis

**FIGURE 10:**
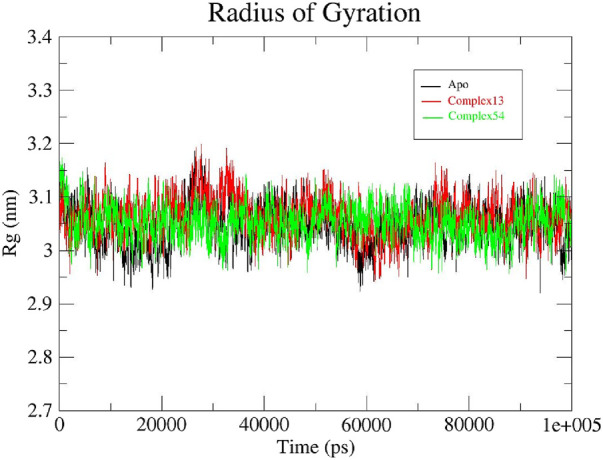
Rg plots of Apo protein (black), Bipinnatin-InlA complex (red) and Epispongiadiol-InlA complex (green).

The radius of gyration (Rg) determined the structural compactness of all the systems under study by measuring the atomic distribution around the center of mass. In other words, Rg examines if the system steadily folds or unfolds during the specific simulation time period (Sharma et al., 2020; Thirumal Kumar et al., 2017; Apicella et al., 2017). Rg plots generated for apo protein, Bipinnatin-InlA and Epispongiadiol-InlA complexes are displayed in Figure 10. It was discovered that both the protein and protein-ligand complexes had relatively same and consistent Rg values over the course of 100 ns trajectory. The average Rg value for all the systems were noted to be 3.05 nm. Therefore, this finding implies that the protein-ligand complexes and apo protein were perfectly compact and in folded condition during the entire 100 ns timescale.

#### 3.4.4 SASA analysis

**FIGURE 11:**
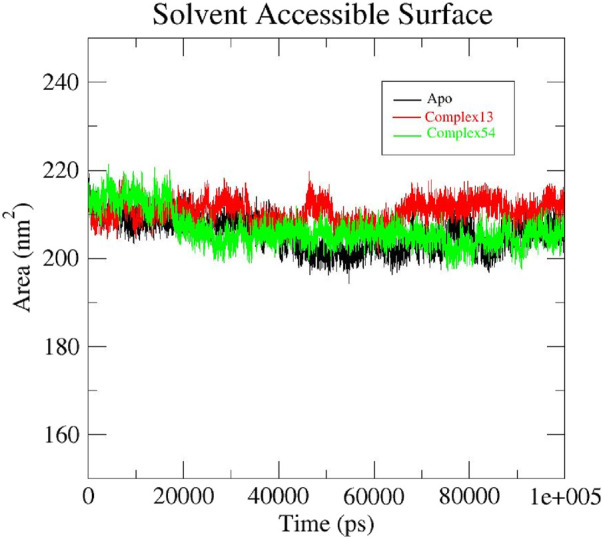
SASA plots of Apo protein (black), Bipinnatin-InlA complex (red) and Epispongiadiol-InlA complex (green).

Solvent accessible surface area (SASA) is a parameter that estimates the area of protein’s surface exposed to solvent. Usually, higher SASA indicate greater exposure of protein residues to water which in turn lead to smaller binding interface area. Hence, a lower SASA value is expected during the MD run which indicates less protein expansion and better stability of the protein structure (Rahman et al., 2021; Saini et al., 2019). Initially, both apo protein and the protein-ligand complexes (ligand 13-InlA and ligand 54-InlA) displayed similar SASA values of 210 nm^2^. As the 100 ns simulation progressed, slight variations in the values were observed for each of the three systems. By the end of 100 ns, both the apo protein and Epispongiadiol-InlA complex had the same SASA value of 205 nm^2^ while the Bipinnatin-InlA complex exhibited a marginally higher value of 215 nm^2^. Thus, the above results suggest that upon the binding of Bipinnatin, the InlA protein had more exposure to solvent when compared to Epispongiadiol. Moreover, the binding of Epispongiadiol had least affected the stability of InlA protein as both the apo form and ligand 54-InlA complex showcased similar SASA values by the end of 100 ns trajectory (Figure 11).

#### 3.4.5 Principal component analysis

**FIGURE 12:**
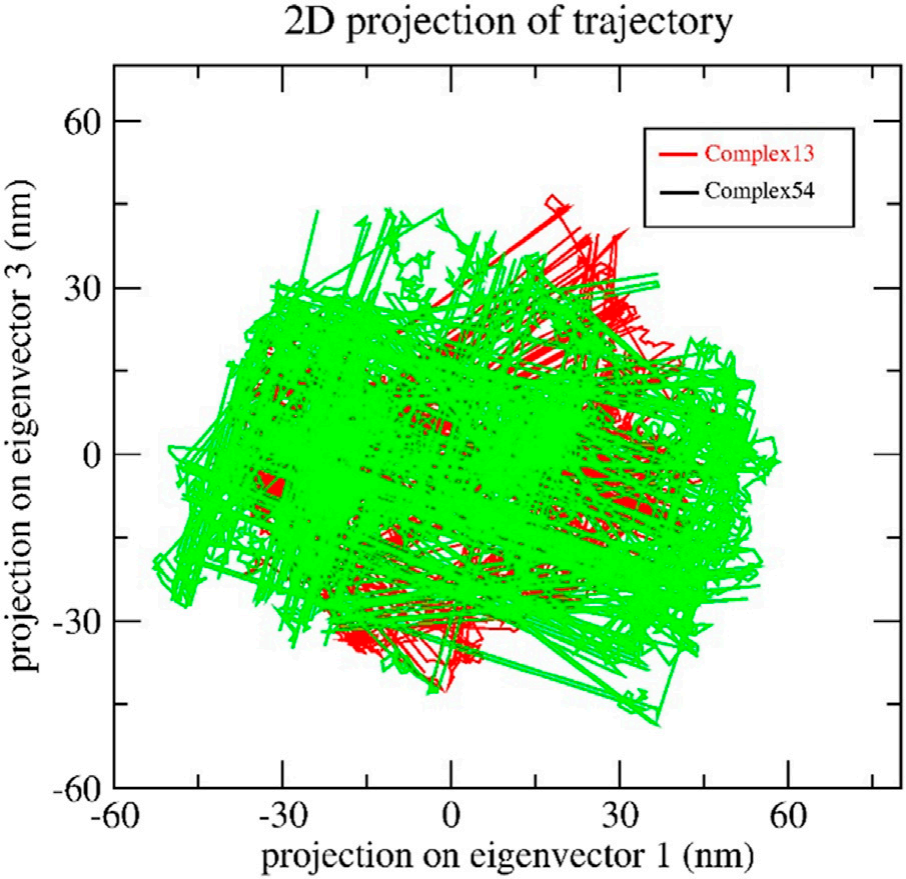
2D trajectory projection of Bipinnatin-InlA (red colour) and Epispongiadiol-InlA (green colour) complexes during MD simulation.

The collective motion of Bipinnatin-InlA and Epispongiadiol-InlA complexes were predicted by PCA. In other words, PCA determines the structural changes that occur in the protein upon ligand binding (Maisuradze et al., 2009). The dynamics of the 2 protein-ligand complexes were estimated with respect to the backbone. The initial principal components (PCs) help in the recognition of dominating motions and also in obtaining a significant portion of variance in the data. In order to understand the motive variations amongst the complex systems, the 2D projection of two eigenvectors (eigenvector 1 in X-axis and eigenvector 3 in the Y-axis) were assessed using the essential dynamics (ED) approach. A system is considered stable if it occupies a small phase space with stable clusters whereas systems that occupies more space and exhibits erratic clusters are categorized as less stable (Tong et al., 2021). Figure 12 indicates that both the complexes almost shared similar conformational spaces suggesting the stable nature of InlA protein upon the binding of Bipinnatin and Epispongiadiol.

## 4 Binding free energy analysis

**FIGURE 13:**
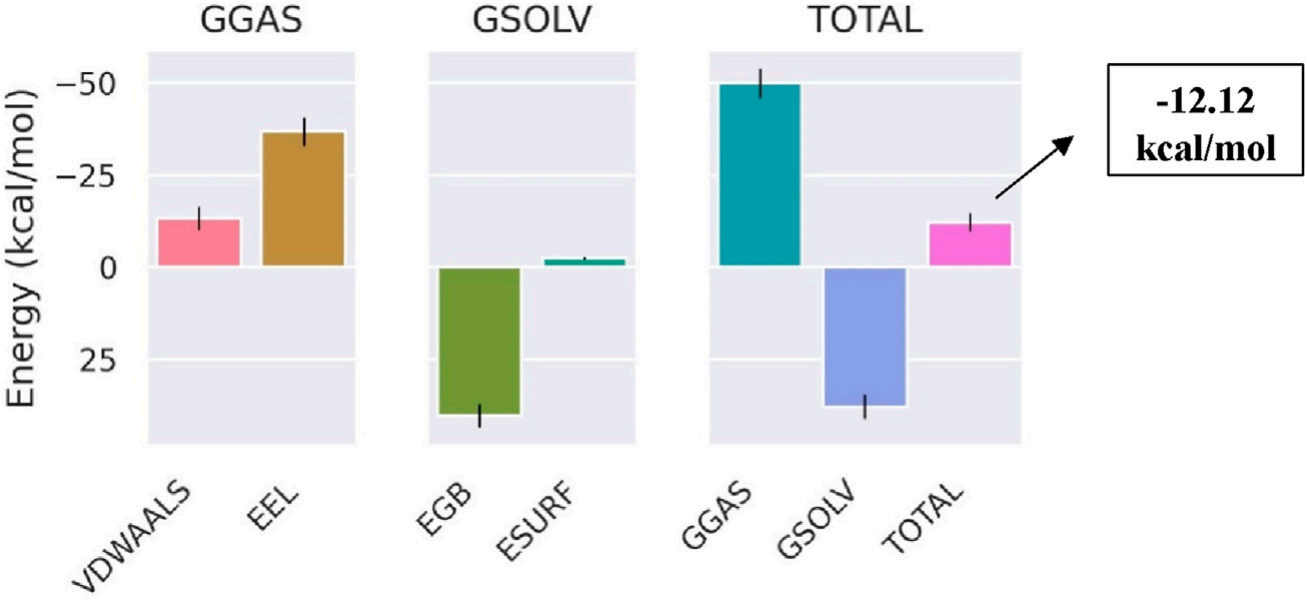
Energy contributions of Bipinnatin-InlA complex.

**FIGURE 14:**
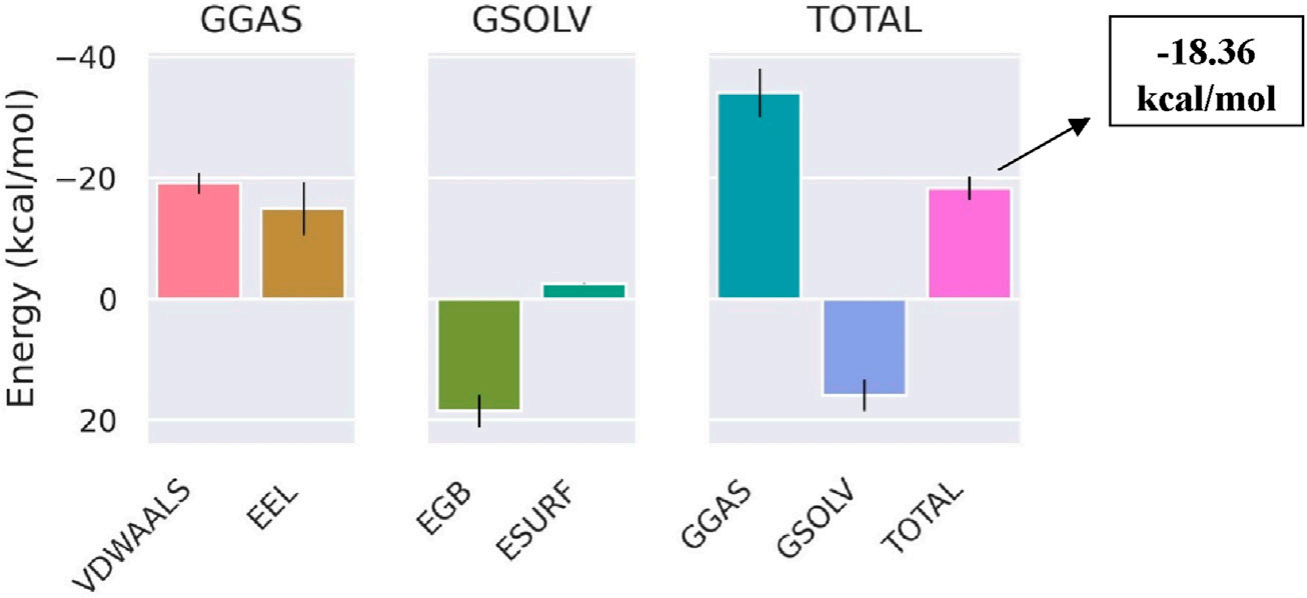
Energy contributions of Epispongiadiol -InlA complex.

The total binding free energy of Bipinnatin-InlA and Epispongiadiol-InlA complexes were computed after 100 ns MD simulation. The overall binding free energy value of Bipinnatin-InlA complex was noted to be −12.12 kcal/mol which indicates the good stability and strong binding interactions between the protein and Bipinnatin (Figure 13). According to the data, the molecular mechanical energy changes in the gas phase (ΔG_Gas_) displayed the highest negative energy value of −49.96 kcal/mol followed by the electrostatic component of internal energy (ΔE_ele_) contributing to a value of −36.76 kcal/mol. In the case of Epispongiadiol-InlA complex, a total binding free energy value of −18.36 kcal/mol was observed which further suggests the strong binding and stable nature of Epispongiadiol-InlA complex (Figure 14). Here, the major contributor to the binding free energy was ΔG_Gas_ (−34.16 kcal/mol) and ΔE_vdW_ (van der Waal’s energy) was the next best contributor with an energy value of −19.15 kcal/mol. Hence, based on the energy data, Epispongiadiol-InlA complex was more stable and had stronger interactions with the protein compared to Bipinnatin due their greater negative binding free energy value. Tables 6, 7 depicts the various energy contributions that resulted in the stable complexes of Bipinnatin and Epispongiadiol with InlA protein.

**TABLE 6:** Binding free energy of Bipinnatin-InlA complex determined using GB calculations.

Energy component	Average (kcal/mol)
ΔE_vdW_	−13.2
ΔE_ele_	−36.76
ΔE_GB_	40.32
ΔE_surf_	−2.48
ΔG_Gas_	−49.96
ΔG_Solv_	37.84
ΔG_bind_	−12.12

**TABLE 7:** Binding free energy of Epispongiadiol-InlA complex determined using GB calculations.

Energy component	Average (kcal/mol)
ΔE_vdW_	−19.15
ΔE_ele_	−15.01
ΔE_GB_	18.43
ΔE_surf_	−2.63
ΔG_Gas_	−34.16
ΔG_Solv_	15.8
ΔG_Total_	−18.36

## 5 Discussion

Listeriosis caused by *L. monocytogenes* is not just a single disease rather a series of illnesses including meningitis, bacteremia, encephalitis or serious pregnancy related infections that can be fatal to those individuals with weakened immune system. According to an earlier study, the Centers for Disease Control and Prevention (CDC) estimated an annual occurrence of 1662 invasive listerial infections in the United States leading to 1520 hospitalizations and 266 deaths (Cartwright et al., 2013). Development of effective medications or antidotes against this disease-causing pathogen continue to be a significant challenge, despite enormous advancements in the medical sector. This failure can be attributed to the bacteria’s growing resistance to antibiotics. The bioactive compounds or phytochemicals found in plants have been explored by several scientists and researchers over the years in an effort to learn more about how these compounds might be able to treat some of the major illnesses caused by different microorganisms.

In the current work, we utilized *in silico* methods to investigate the possible antibacterial effect of terpenes on one of the virulence proteins of *L. monocytogenes*, InlA. Many research studies have reported a range of therapeutic properties of terpenes including anticancer, antiinflammatory, antiviral, antidiabetic, antiplasmodial and antimicrobial activities (Tetali, 2019). For example, researchers have discovered terpenes and its derivatives to be potential inhibitors of SARS-CoV-2 proteases as well as a promising natural antagonists of cancer through *in silico* studies (Diniz et al., 2021; Muhseen and Li, 2019). In our previously published work, the inhibitory effect of terpenoids (derived from terpenes) on another virulence protein of *L. monocytogenes* were predicted utilizing computational methods (Deepasree and Subhashree, 2023). Computational methodologies have proven to be very beneficial in pharmaceutical research because of their capacity to identify and generate novel, potent drugs, especially through the use of molecular docking and molecular dynamic simulation approaches. Every terpene chosen for this investigation complied with “Lipinski’s rule of five” and when these compounds were docked against the target protein InlA, it became evident that Bipinnatin and Epispongiadiol had the highest negative binding affinity values (−9.5 kcal/mol), which made them the most potent drug-like compounds. This work stands out for offering innovation as there are only very few reports linked to the aforementioned compounds. Selecting only the top ten compounds with the best docking scores, we proceeded to investigate the pharmacokinetic properties and also predicted the possible biological activities that these ten compounds might exhibit. The drugs chosen for analysis provided acceptable ADMET results for the compounds and also found that all the compounds with an exception of Inuchinenolide C, Furodysinin and Axinysone C displayed antibacterial activity which supports the goal of the study. Finally, the two compounds with the best docking scores were subjected to MD simulation for 100 ns and the results obtained suggests Epispongiadiol to be the best compound over Bipinnatin due to its greater structural stability. However, the aforementioned computational approaches have certain limitations as these methods don’t completely capture the cellular environments and other crucial interactions. In addition, the exact mechanism of action or resistance mechanisms may not be predicted by computational studies alone. Thus, preclinical and clinical investigations would be required to explore the *in silico* predictions in biological models for further therapeutic applications.

## 6 Conclusion

This study aimed to understand the potential of 80 terpenes in inhibiting one of the major virulence protein, Internalin A of the pathogen *L. monocytogenes*. Among the terpenes, Bipinnatin and Epispongiadiol were discovered to be the potential drug-like candidates that could be targeted against InlA due to their good binding affinity value of −9.5 kcal/mol. Although both the compounds possessed antibacterial activity upon biological activity evaluation, Bipinnatin belongs to a category of compounds that induces less toxicity only at lower doses according to ADMET analysis while Epispongiadiol is non-toxic even at higher doses. Results of the MD simulation for 100 ns revealed Epispongiadiol to be better than Bipinnatin due to their overall structural stability despite both the compounds having identical docking scores. The MM/GBSA binding free energy analysis confirmed the stable nature and stronger interaction of Epispongiadiol with the virulence protein. Hence, based on the *in silico* investigation, this work reports for the first time that Epispongiadiol could be a possible antibacterial agent that can be directed against InlA protein. Future studies are intended for the *in vivo/in vitro* validation with the best compound identified from this work.

## Data Availability

The datasets presented in this study can be found in online repositories. The names of the repository/repositories and accession number(s) can be found in the article/supplementary material.
